# Levulinic Acid-Inducible and Tunable Gene Expression System for *Methylorubrum extorquens*

**DOI:** 10.3389/fbioe.2021.797020

**Published:** 2021-12-15

**Authors:** Chandran Sathesh-Prabu, Young Shin Ryu, Sung Kuk Lee

**Affiliations:** ^1^ School of Energy and Chemical Engineering, Ulsan National Institute of Science and Technology (UNIST), Ulsan, South Korea; ^2^ Department of Energy Engineering, Ulsan National Institute of Science and Technology (UNIST), Ulsan, South Korea

**Keywords:** *Methylorubrum extorquens* AM1, levulinic acid, promoter, inducible expression, C1-biorefinery

## Abstract

*Methylorubrum extorquens* AM1 is an efficient platform strain possessing biotechnological potential in formate- and methanol-based single carbon (C1) bioeconomy. Constitutive expression or costly chemical-inducible expression systems are not always desirable. Here, several glucose-, xylose-, and levulinic acid (LA)-inducible promoter systems were assessed for the induction of green fluorescent protein (GFP) as a reporter protein. Among them, the LA-inducible gene expression system (HpdR/P_*hpdH*_) showed a strong expression of GFP (51-fold) compared to the control. The system was induced even at a low concentration of LA (0.1 mM). The fluorescence intensity increased with increasing concentrations of LA up to 20 mM. The system was tunable and tightly controlled with meager basal expression. The maximum GFP yield obtained using the system was 42 mg/g biomass, representing 10% of the total protein content. The efficiency of the proposed system was nearly equivalent (90%–100%) to that of the widely used strong promoters such as P_*mxaF*_ and P_*L/O4*_. The HpdR/P_*hpdH*_ system worked equally efficiently in five different strains of *M. extorquens*. LA is a low-cost, renewable, and sustainable platform chemical that can be used to generate a wide range of products. Hence, the reported system in potent strains of *M. extorquens* is highly beneficial in the C1-biorefinery industry to produce value-added products and bulk chemicals*.*

## Introduction

Microbial utilization of single-carbon (C1) compounds as a feedstock to produce fuels, chemicals, and other value-added products is an ideal strategy to combat the depletion of fossil fuels and environmental pollution (Humphreys and Minton, 2018). C1 gases such as CO and CO_2_ are abundant and low-cost waste products generated by various industries such as petroleum refining, thermal power plants, steel mills, and landfills (Humphreys and Minton, 2018; Hwang et al., 2020). Although several studies have reported the utilization of CO as an energy and/or carbon source for the production of fuels and chemicals (Rittmann et al., 2015; Kim et al., 2016), the direct conversion of CO-containing gaseous waste into value-added products is challenging owing to its high toxicity to most living organisms. At the same time, reduced C1 compounds such as formate and methanol can be synthesized through the electrochemical reduction of CO_2_ or the hydration of synthesis gas (a mixture of CO and H_2_) at a relatively low cost (Agarwal et al., 2011; Ganesh, 2014; Hwang et al., 2020). These compounds are considered sustainable and eco-friendly microbial feedstocks, as well as alternatives to sugar-based raw materials for industrial fermentation (Belkhelfa et al., 2019; Yoon et al., 2021).

Among the methylotrophic microorganisms, the pink-pigmented facultative methylotrophic alpha-proteobacterium *Methylorubrum extorquens* AM1 is widely used as a catalyst; it can use both C1 (formate and methanol) and multi-carbon compounds such as succinate (C4), pyruvate (C3), and acetate (C2) as the sole carbon and energy sources (Peyraud et al., 2012; Schneider et al., 2012; Ochsner et al., 2015; Green and Ardley, 2018). Furthermore, the availability of the complete genomic sequence (Vuilleumier et al., 2009), metabolic models (Peyraud et al., 2011), and the basic set of genetic tools for overexpression, knockout, and transposon mutagenesis (Marx and Lidstrom, 2001; Ochsner et al., 2015) makes the strain an attractive host for biotechnological applications. Strain AM1 employs a number of interlocked metabolic processes for carbon assimilation, such as C1 pathways, the serine cycle, the TCA cycle, the ethylmalonyl-CoA (EMC) pathway, the poly-3-hydroxybutyrate (PHB) cycle, and gluconeogenesis (Anthony, 2011; Peyraud et al., 2011). These processes are connected by overlapping metabolites and enzymatic reactions (Peyraud et al., 2011). When cultivated on media containing formate or methanol, the metabolic flux largely goes through the tetrahydrofolate, serine, and EMC pathways, generating a stable supply of acetyl-CoA, which could serve as a precursor for the production of value-added chemicals in engineered *M. extorquens* AM1 strains (Peyraud et al., 2011; Zhang et al., 2019; Hwang et al., 2020). This strain has been engineered to produce a variety of value-added chemicals or biofuels such as PHB (Hwang et al., 2020; Yoon et al., 2021), polyhydroxyalkanoate terpolymers (Orita et al., 2014), 1-butanol (Hu and Lidstrom, 2014), 3-hydroxypropionic acid (3-HP) (Yang et al., 2017), mevalonic acid (Zhu et al., 2016), butadiene precursor (Yang et al., 2018), terpenoids (Sonntag et al., 2015a), mesaconic acids (Sonntag et al., 2015b), crotonic acids (Schada Von Borzyskowski et al., 2018), and itaconic acids (Lim et al., 2019) from formate or methanol.

The overexpression of genes in *M. extorquens* AM1 is a challenging task, as the commonly used promoters (such as λP_*L*_ and λP_R_) are inactive, and the P_*lac*_ promoter and its derivatives confer weak or leaky expression in *M. extorquens* (Marx and Lidstrom, 2001; Chubiz et al., 2013). These data indicate a lack of dynamic control of gene expression in *M. extorquens*. The strong native P_*mxaF*_ promoter, which drives the expression of methanol dehydrogenase in *M. extorquens*, has almost exclusively been used for constitutive gene expression. A set of constitutive promoters (P_*mxaF*_, P_*coxB*_, P_*fumC*_, and P_*tuf*_) of different strengths has been reported for the expression of genes in *M. extorquens* AM1 (Schada Von Borzyskowski et al., 2015). In general, uncontrolled high-level expression or constitutive expression systems are not always desirable because they are detrimental to cells due to uncontrolled production of target protein(s) and structural instability (Lee and Keasling, 2005; Chaves et al., 2020). Moreover, cumate- or anhydrotetracycline-inducible hybrid promoters (P_*R/cmtO*_ and P_*R/tetO*_) with low basal expression and high expression upon induction have been developed (Chubiz et al., 2013). Recently, Carrillo et al. (2019) introduced a set of isopropyl β-d-1-thiogalactopyranoside (IPTG)-inducible promoters that were strong, tight, and controllable, even exceeding the promoter elements available thus far. However, IPTG is not suitable on an industrial scale because of its toxicity and cost (Makrides, 1996; Browning et al., 2017). Thus, the use of inexpensive renewable substrates as inducers could be a feasible strategy for the large-scale production of biochemicals (Sathesh-Prabu et al., 2021).

Therefore, to expand the biotechnological potential of *M. extorquens* AM1 in formate- and methanol-based C1 bioeconomy, we aimed to develop an efficient tunable gene expression system with low-cost renewable substrates such as glucose, xylose, and levulinic acid (LA). These substrates can be used as starting materials to produce a wide range of chemicals (Cha et al., 2020; Hayes and Becer, 2020; Sathesh-Prabu et al., 2020). In the present study, we characterized the LA-inducible expression system for the induction of the expression of green fluorescent protein (GFP) as a quantitative reporter of promoter activity.

To our knowledge, this is the first report describing the characterization of an LA-inducible and tunable promoter system for synthetic biological applications in *M. extorquens* AM1.

## Materials and Methods

### Microbial Strains, Plasmids, and Culture Conditions

The wild-type *M. extorquens* AM1 strain was used to analyze the efficiency of the inducible promoter systems. The *Escherichia coli* strain DH10B was used for cloning. All plasmids and strains used in this study are listed in Supplementary Table S1. *M. extorquens* AM1 was cultivated in minimal salt medium (MSM) at 30°C under aerobic conditions, with agitation in an orbital incubator shaker at 200 rpm. The MSM contained 1.62 g NH_4_Cl, 0.2 g MgSO_4_, 2.21 g K_2_HPO_4_, 1.25 g NaH_2_PO_4_.2H_2_O, 15 mg Na_2_EDTA.2H_2_O, 4.5 mg ZnSO_4_.7H_2_O, 0.3 mg CoCl_2_.6H_2_O, 1 mg MnCl_2_.4H_2_O, 1 mg H_3_BO_3_, 2.5 mg CaCl_2_, 0.4 mg of Na_2_MoO_4_.2H_2_O, 3 mg FeSO_4_7H_2_O, 0.3 mg CuSO_4_.5H_2_O, and 4 g succinate per liter. *E. coli* was cultured in a lysogeny broth (5 g yeast extract, 10 g peptone, and 10 g NaCl per liter) at 37°C, with agitation in a shaker incubator at 200 rpm. Tetracycline (10 μg/ml) was added to the growth medium to cultivate all the recombinant strains. All chemicals were purchased from Sigma-Aldrich (St. Louis, MO, USA). LA was neutralized with 10 N NaOH and sterilized prior to use.

### Construction of Inducible Expression Systems

Restriction enzymes, the Q5 high-fidelity DNA polymerase, and the Gibson assembly cloning kit were purchased from New England Biolabs (Ipswich, MA, USA) and used for cloning and plasmid construction. Five different promoter systems for the *M. extorquens* AM1 strain, including glucose-inducible HexR/P_*zwf1*_, LA-inducible LvaR/P_*lvaA*_, 3HP- and LA-inducible HpdR/P_*hpdH*_ and MmsR/P_*mmsA*_, and xylose-inducible XutR/P_*xutA*_, were evaluated. In our previous study (Sathesh-Prabu et al., 2021), we evaluated the efficiency of these inducible expression systems in *Pseudomonas putida* KT2440 by constructing promoter candidates in the pPROBE_eGFP^+^ plasmid (pBBR1-*ori*, Km^R^: a broad-host-range expression vector) (Kim et al., 2019), which served as a template for PCR amplification in the present study. To construct the inducible expression systems in *M. extorquens* AM1, the fragments containing the coding sequence of the transcription regulator of interest, their respective promoter sequences, and eGFP^+^ were generated through PCR using the respective plasmids as templates. The fragments were then cloned into the *Xba*I/*Bam*HI double-digested expression vector pCM110_P_*mxaF*__Fdh1, a derivative of the pCM110 plasmid (Marx and Lidstrom, 2001), *via* the Gibson assembly to generate the following plasmids: pMHRZ_eGFP^+^, pMLRL_eGFP^+^, pMHRH_eGFP^+^, pMMRM_eGFP^+^, and pMXRX_eGFP^+^, harboring the HexR/P_*zwf1*_, LvaR/P_*lvaA*_, HpdR/P_*hpdH*_, MmsR/P_*mmsA*_, and XutR/P_*xutA*_ systems, respectively. In addition, to compare the efficiency of the constructed systems, LacI/P_*L/O4*_ (Carrillo et al., 2019), the strongest inducible promoter system reported to date in *M. extorquens* AM1, was similarly constructed in the pCM110 plasmid, thereby generating pMPLO4_eGFP^+^. The LacI/P_*L/O4*_ promoter was adapted from the pCM110_P_*L/O4*_ plasmid. The oligonucleotides used for the PCR amplification of each promoter system are listed in Supplementary Table S2.

### Construction of Recombinant Strains

The constructed plasmids were introduced into the electrocompetent cells of *M. extorquens* AM1. The electrocompetent cells of *M. extorquens* AM1 were prepared as previously described (Toyama et al., 1998). The constructed plasmids were transformed into electrocompetent cells *via* electroporation (0.1-cm gap cuvette at a voltage of 1.8 kV) using a MicroPulser electroporator (Bio-Rad, Hercules, CA, USA). Subsequently, ice-cold sterile nutrient broth (3 g beef extract and 5 g peptone per liter) was added to the cuvette. The cell suspension was transferred into a test tube and incubated at 30°C and 200 rpm for 3 h prior to being spread onto MSM with 1.5% agar, 0.4% succinate, and tetracycline (10 μg/ml).

### Promoter Expression Assay

The GFP fluorescence intensity of the recombinant strains was estimated using their respective inducers. For the primary evaluation of all the inducible expression systems constructed in this study, the recombinant strains were first cultured in MSM until the late exponential phase and then subcultured [initial optical density at 600 nm (OD_600_) set to 0.1] in 5 ml of fresh MSM medium. After the strains were incubated for at least 12 h (OD_600_ ≈ 0.5–0.7), they were induced with 10 mM solutions of their respective inducers (glucose, xylose, LA, or 3-HP) or 1 mM IPTG solution. Thereafter, 180 µl of culture was collected at 24 h after induction and inoculated into a clear bottom Corning 96-well plate to measure the GFP fluorescence intensity (a gain of 30 with an excitation wavelength at 485 nm and emission wavelength at 535 nm) of the constructed systems in a microplate fluorescence reader (Infinite F200 PRO; Tecan, Grődig, Austria). Subsequently, the culture was diluted appropriately with phosphate-buffered saline (pH 7.2), and flow cytometric analysis of GFP fluorescence was performed using fluorescence-activated cell sorting (FACSCalibur Flow Cytometer; BD Biosciences, San Jose, CA, USA). Approximately 2 × 10^5^ cells were analyzed per sample. The fluorescence intensity was normalized based on the OD_600_ of the culture.

### Optimization of LA Induction

To determine the optimal time for the induction of the HpdR/P_*hpdH*_ system, following the preparation of the culture as described above, LA (10 mM) was added at 6-h intervals from the initial inoculation (OD_600_ ≈ 0.1) for 24 h. Samples were collected every 6 h to measure the GFP fluorescence intensity for 48 h. To analyze the degradation of the added LA, residual concentrations of LA in the supernatant free of cells from the culture media of recombinant strains at 0, 24, and 48 h after LA addition (10 mM) were measured using high-performance liquid chromatography (HPLC) (Shimadzu HPLC station equipped with a refractive index detector and an SIL-20A auto-sampler, all from Shimadzu, Kyoto, Japan). The samples were eluted through a 4.6-mm  150-mm, 5-μm Zorbax SB-Aq column (Agilent, Santa Clara, CA, USA) at 40°C using 25 mM ammonium formate (pH 2.0) as the mobile phase, at a flow rate of 1 ml/min. The cell density was monitored with the OD_600_ using a Biochrom Libra S22 spectrophotometer (Biochrom, Cambridge, UK). An OD_600_ of 1.0 was equivalent to 0.278 g/L dry cell weight (Skovran et al., 2010).

To determine the optimal LA dosage, different concentrations (0.1, 0.25, 0.5, 1, 2, 4, 8, 10, 20, 40, 50, and 100 mM) of LA were added as a single dose at 0 h. Samples were collected at 12 and 24 h after induction to measure the GFP fluorescence intensity.

### **Qualitative and Quantitative Measurement of eGFP** ^**+**^

Following the optimization of time and concentration of LA for the induction of the HpdR/P_*hpdH*_ system, the expression of eGFP^+^ was induced with 10 mM of LA, and the cells were harvested after 24 h of induction. The cell pellet was resuspended in ice-cold cell lysis buffer (50 mM MOPS, pH 8.0, 10 mM imidazole), and cell lysates were prepared by ultrasonication (35% amplitude, 10 s ON/OFF, total sonication ON time = 6 min) using a programmable ultrasonic processor (VCX 130; Sonics, Newton, CT, USA). Protein concentration was determined with the Bradford assay reagent using bovine serum albumin (BSA) as a standard. The samples were then diluted in sodium dodecyl sulfate-polyacrylamide gel electrophoresis (SDS-PAGE) sample loading buffer, and 5 µg per well was loaded onto a 15% polyacrylamide gel. The protein bands were visualized by staining with Coomassie Brilliant Blue R-250. The concentration of eGFP^+^ in the sample was calculated based on a linear relationship between known concentrations of purified GFP (Abcam, Cambridge, MA, USA) and the fluorescence units, according to the manufacturer’s protocol.

## Results

### Evaluation of the Inducible Expression Systems

**FIGURE 1:**
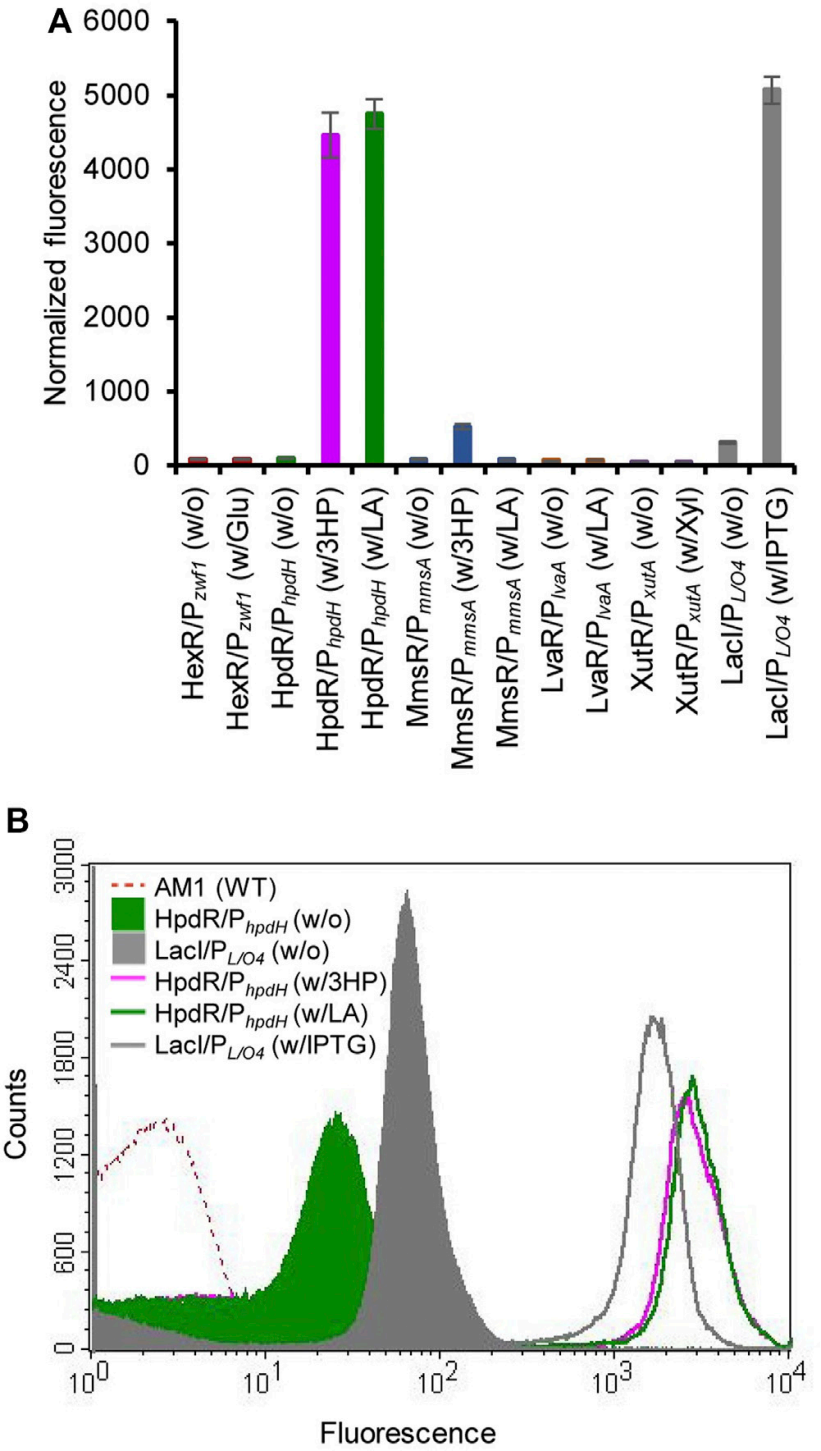
Evaluation of various inducible expression systems in *Methylorubrum extorquens* AM1. **(A)** Normalized green fluorescent protein (GFP) fluorescence intensity of the various inducible expression systems (HexR/P_*zwf1*_, LvaR/P_*lvaA*_, HpdR/P_*hpdH*_, MmsR/P_*mmsA*_, XutR/P_*xutA*_, and LacI/P_*L/O4*_) induced by their respective inducers. *System name (w/o)* and *System name (w/x)* indicate the absence and presence of the respective inducers, respectively. The systems were induced with 10 mM solutions of the respective inducers (glucose, xylose, LA, or 3-HP) or 1 mM IPTG. The HpdR/P_*hpdH*_ system showed the maximum expression upon induction either by LA or 3-HP. **(B)** Flow cytometric analysis of the HpdR/P_*hpdH*_ system (strain MHRH01) and the LacI/P_*L/O4*_ system (strain MPLO4) induced by 10 mM LA and 1 mM IPTG, respectively. Data represent the mean ± SD of the three biological replicates. *Glu*, glucose; *LA*, levulinic acid; *3HP*, 3-hydroxypropionic acid; *Xyl*, xylose; *IPTG*, isopropyl β-d-1-thiogalactopyranoside.

The constructed plasmids pMHRZ_eGFP^+^, pMLRL_eGFP^+^, pMHRH_eGFP^+^, pMMRM_eGFP^+^, pMXRX_eGFP^+^, and pMPLO4_eGFP^+^ were individually transformed into *M. extorquens* AM1 to generate the MRHZ01, MLRL01, MHRH01, MMRM01, MXRX01, and MPLO4 strains, respectively. The inducibility of the constructed expression systems was analyzed by measuring the GFP fluorescence of the strains cultivated in MSM supplemented with glucose (MRHZ01), LA or 3-HP (MLRL01, MHRH01, and MMRM01), xylose (MXRX01), or IPTG (MPLO4). The results showed that the HpdR/P_*hpdH*_ and MmsR/P_*mmsA*_ systems significantly (*p* < 0.05) increased the fluorescence intensity when induced by 3-HP or LA and 3-HP, respectively, compared to that in the control (Figure 1A). The GFP expression level of HpdR/P_*hpdH*_ induced by LA was similar to that of HpdR/P_*hpdH*_ induced by 3-HP (*p* > 0.05), a known inducer of the HpdR/P_*hpdH*_ system (Zhou et al., 2015; Hanko et al., 2017; Sathesh-Prabu et al., 2021). In contrast, the MmsR/P_*mmsA*_ system was not induced by LA (Figure 1A).

Other systems (HexR/P_*zwf1*_, LvaR/P_*lvaA*_, and XutR/P_*xutA*_) did not show any comparable fluorescence intensity upon induction when compared to that of the control condition. Flow cytometric analysis of the inducible promoter systems (HexR/P_*zwf1*_, LvaR/P_*lvaA*_, MmsR/P_*mmsA*_, and XutR/P_*xutA*_) is shown in Supplementary Figure S1. In the present study, we compared the efficiency of the constructed expression systems with IPTG-inducible LacI/P_*L/O4*_, demonstrating a maximum strength of 157% relative to the conventional P_*mxaF*_ promoter. HpdR/P_*hpdH*_ (strain MHRH01) induced with LA (10 mM) achieved 94% efficiency of the LacI/P_*L/O4*_ system induced with IPTG (1 mM). In contrast, MmsR/P_*mmsA*_ (strain MMRM01) showed 10% of the efficiency exhibited by LacI/P_*L/O4*_. As the efficiency of the HpdR/P_*hpdH*_ (strain MHRH01) system was similar to that of the strongest IPTG-inducible expression (LacI/P_*L/O4*_) system (*p* > 0.05), the HpdR/P_*hpdH*_ system was selected for further study. Notably, without the inducer, the HpdR/P_*hpdH*_ system was tightly controlled (no leaky expression) (Figure 1B and Supplementary Figure S2). The HpdR/P_*hpdH*_ system was at least threefold less leaky than the uninduced LacI/P_*L/O4*_. Furthermore, the HpdR/P_*hpdH*_ system was characterized using LA as the inducer.

### **Optimization of HpdR/P**_***hpdH***_ **Induction**

**FIGURE 2:**
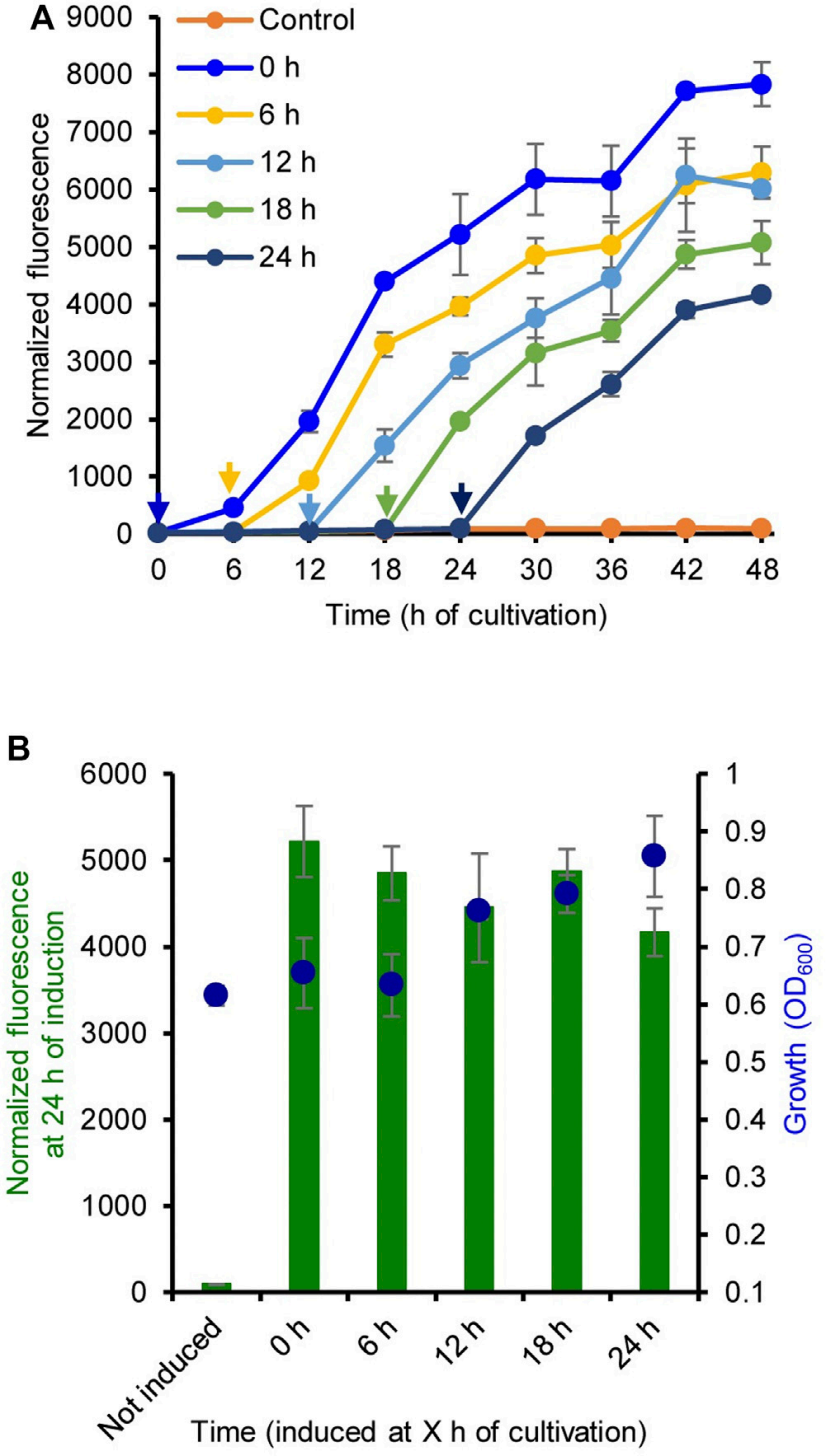
Optimization of the induction time of levulinic acid (LA) addition. **(A)** Normalized green fluorescent protein (GFP) fluorescence intensity of the strain MHRH01 induced by 10 mM LA. LA was added at different time points during cultivation. The *arrows* indicate the time of LA addition. **(B)** Normalized fluorescence intensity of the strain MHRH01 measured at 24 h after induction. Data represent the mean ± SD of three biological replicates.

The induction time for LA was also evaluated. Figure 2A shows the GFP fluorescence intensity of strain MHRH01 (HpdR/P_*hpdH*_) induced by LA (10 mM) at 0, 6, 12, 18, and 24 h of cultivation. When analyzing the fluorescence intensity of the strains at 24 h of induction, the addition of LA at different time points did not show any significant difference (*p* > 0.05) in the expression of eGFP^+^ (Figure 2B). This property allows the addition of LA during the early or exponential growth phase, thereby avoiding the requirement of constant monitoring of cell growth to ensure that the inducer, such as IPTG, is added at the optimal cell density (Hu et al., 2015; Briand et al., 2016). The addition time of IPTG was critical for IPTG-inducible expression systems. IPTG induction during the early exponential growth phase was more efficient than that in the middle exponential growth phase or late exponential growth phase (Briand et al., 2016). Our system reported here eases the addition of an inducer to the culture medium.

**FIGURE 3:**
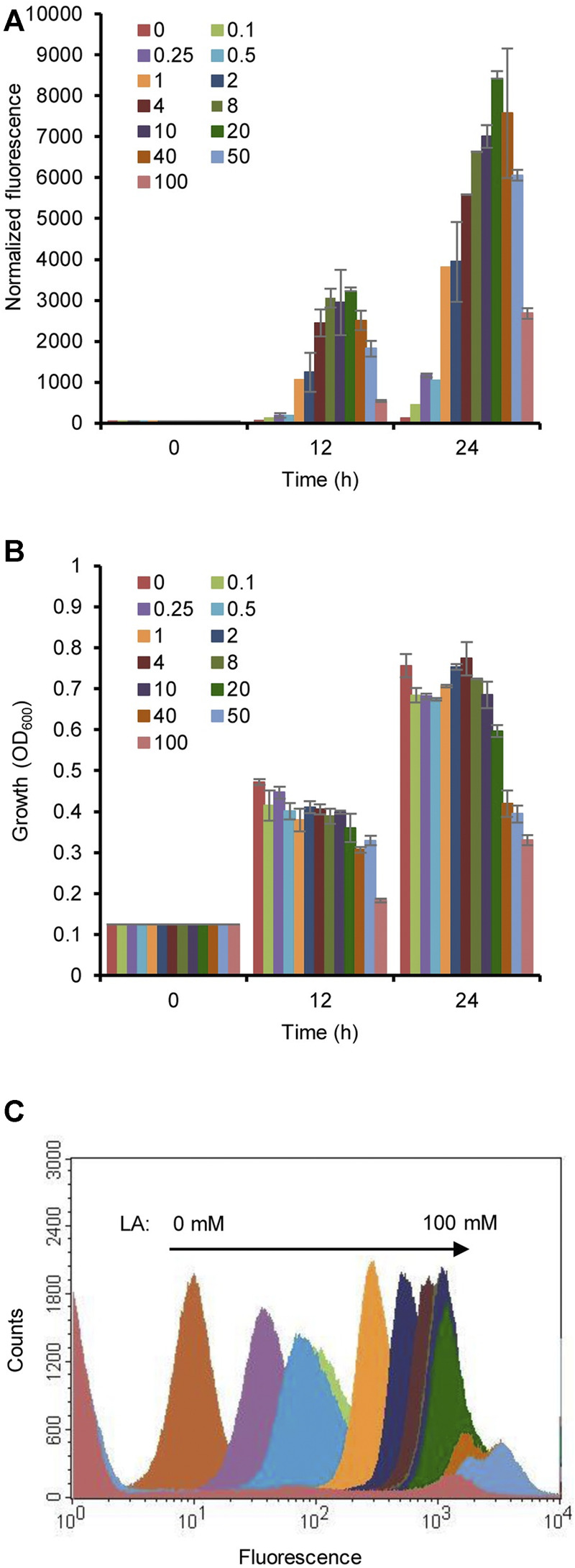
Optimization of the concentration of levulinic acid (LA). **(A)** Normalized green fluorescent protein (GFP) fluorescence intensity of the strain MHRH01 induced by different concentrations of LA. LA was added at 0 h of cultivation. The intensity increased with increasing concentrations of LA up to 20 mM of LA. **(B)** Measurement of the cell growth of strain MHRH01 induced by different concentrations of LA. Beyond 20 mM LA, growth retardation was observed. **(C)** Flow cytometric analysis of strain MHRH01 induced by different concentrations of LA. The samples were collected after 24 h of induction for analysis. MHRH01 showed tight control and homogeneity (cell populations with similar expression levels) of the HpdR/P_*hpdH*_ expression system up to 20 mM of LA.

To optimize the concentration of LA, the induction of the HpdR/P_*hpdH*_ system was analyzed by estimating the fluorescence intensity of the strain MHRH01 with different concentrations of LA (0.1–100 mM). The fluorescence intensity of strain MHRH01 increased with increasing concentrations of LA (Figure 3A) up to 20 mM LA, demonstrating the dose-dependent expression of the reporter protein. The system was induced even at a low concentration of LA (0.1 mM), showing a fourfold stronger fluorescence intensity than that observed in the control. However, the intensity decreased beyond 20 mM LA. Approximately 10%, 28%, and 68% decreased intensities were observed at 40, 50, and 100 mM LA, respectively, compared to the fluorescence intensity observed at 20 mM LA (Figure 3A). The decreased efficiency was attributed to the reduced cell growth of the strain when cultivated in the presence of more than 20 mM LA (Figure 3B). A maximum 1.5-fold reduction in cell growth was observed with 50 mM LA. Flow cytometric analysis of HpdR/P_*hpdH*_ in MHRH01 induced by different concentrations of LA showed tight control and homogeneity (cell populations with similar expression levels) of the expression system up to 20 mM of LA (Figure 3C).

**FIGURE 4:**
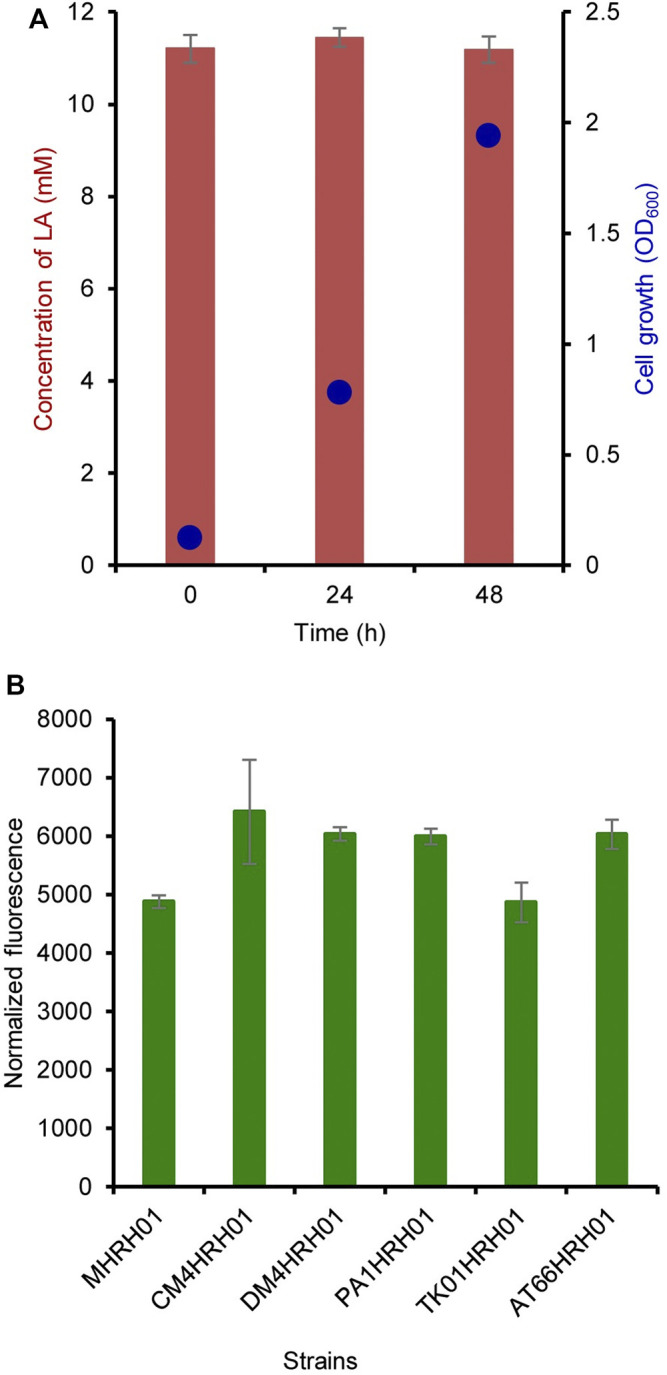
Non-degradation of levulinic acid (LA) and strain independence of the HpdR/P_*hpdH*_ system. **(A)** Residual concentration of LA measured at 0, 24, and 48 h after its addition. The concentration of LA did not reduce over the cultivation period. **(B)** Strain independence of the HpdR/P_*hpdH*_ system. The normalized green fluorescent protein (GFP) fluorescence intensities of the various *Methylorubrum* strains (CM4, DM4, PA1, TK0001, and ATCC55366) harboring pMHRH-eGFP^+^ were measured. The system showed similar levels of expression in all the strains induced with 10 mM LA. Data represent the mean ± SD of three biological replicates.

The residual concentration of LA was measured at 0, 24, and 48 h of cultivation. LA (10 mM) was added at 0 h of cultivation. As shown in Figure 4A, LA was not consumed by the strain MHRH01 even after sufficient cell growth and showed OD_600_ values of 0.125 and 1.94 at 0 and 48 h, respectively.

The plasmid pMHRH_eGFP^+^ was transformed into other *Methylorubrum* strains, such as CM4 (KCTC 32005), DM4 (DSM 6343), PA1 (DSM 23939), TK0001 (DSM1337), and ATCC55366, to evaluate the strain independence of the HpdR/P_*hpdH*_ system. The system showed a similar level of expression in all strains upon induction with 10 mM LA (Figure 4B). The strain independence of the constructed expression system would be highly useful for a wide range of applications in different strains.

### Quantitation of GFP

**FIGURE 5:**
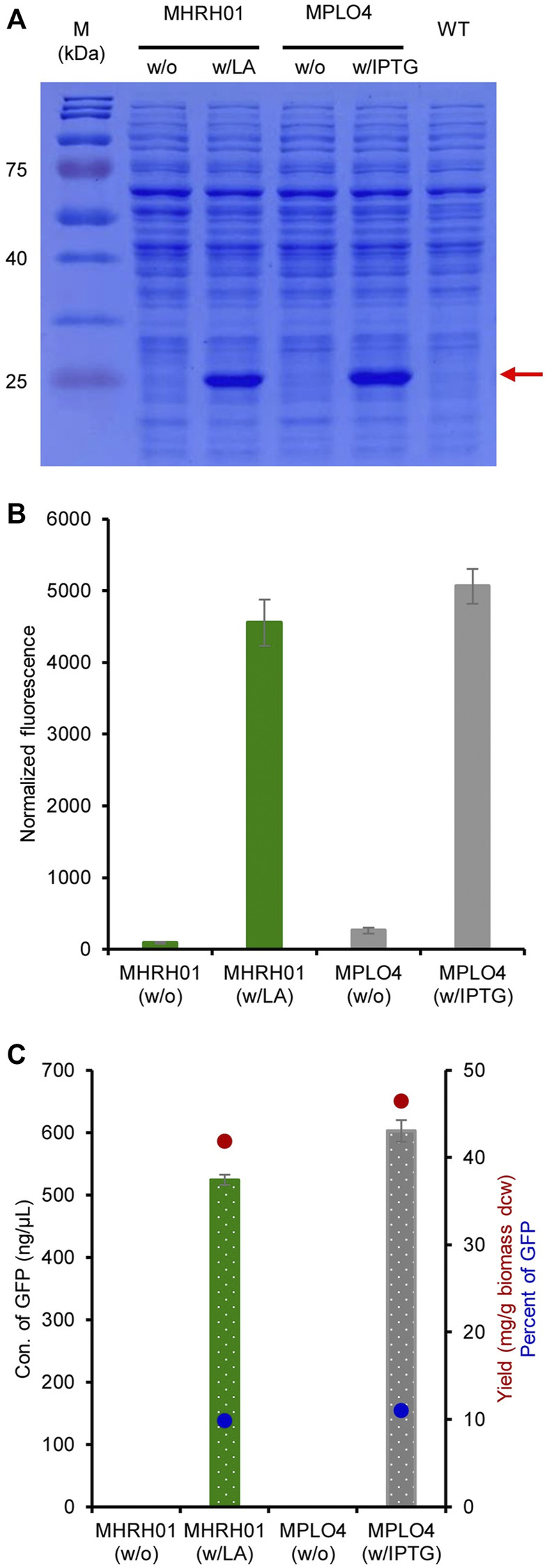
Efficiency of the HpdR/P_*hpdH*_ system in the production of green fluorescent protein (GFP). **(A)** SDS-PAGE analysis of GFP. Strains MHRH01 and MPLO4 showed a strong expression of eGFP^+^ (27 kDa), indicated by the *red arrow*, upon induction with 10 mM LA and 1 mM IPTG, respectively. **(B)** Maximum normalized fluorescence intensity of strains MHRH01 and MPLO4 induced with 10 mM LA and 1 mM IPTG, respectively. **(C)** Quantitation of GFP. Strains MHRH01 and MPLO4 efficiently produced eGFP^+^ with yields of 42 and 46 mg/g of biomass (dry cell weight), representing around 10% and 11% of the total protein content, respectively. Data represent the mean ± SD of three biological replicates.

The efficiency of the constructed system for producing GFP was analyzed both qualitatively and quantitatively. The SDS-PAGE results shown in Figure 5A reveal a strong expression of eGFP^+^ (27 kDa) by the strain MHRH01 induced by 10 mM LA. The strain did not produce eGFP^+^ (no protein bands were visualized) in the absence of LA. The normalized GFP fluorescence intensities of strains MHRH01 and MPLO4 are shown in Figure 5B. In addition, the concentration of GFP produced by strain MHRH01 was measured. The strains MHRH01 and MPLO4 efficiently produced eGFP^+^, with yields of 42 and 46 mg/g of biomass (dry cell weight), representing approximately 10% and 11% of the total protein content, respectively (Figure 5C). The efficiency of the LA-inducible system was almost equal (90%) to that of the IPTG-inducible system (*p* > 0.05).

## Discussion

*Methylorubrum extorquens* AM1 has been widely used in methylotrophic studies and has attracted great interest from the scientific community because of its versatile metabolism of C1 compounds. In the present study, we developed an LA-inducible expression system for use in *M. extorquens* AM1. The efficiency of the system was evaluated by inducing the expression of the fluorescent reporter gene (e*gfp* ^+^) as a quantitative reporter of relative promoter activity (Born and Pfeifer, 2019; Sathesh-Prabu et al., 2021). The strength of the GFP signal directly correlates with the promoter activity. GFP has been widely used as a model protein to study heterologous protein expression in different kinds of microorganisms, including *M. extorquens* AM1 (Bélanger et al., 2004; Choi et al., 2006), because of its unique characteristics, such as no requirement of additional substrates and/or cofactors and enzymes, no cell lysis, sensitivity, and the short analysis time (Lissemore et al., 2000; Born and Pfeifer, 2019).

In our previous study, we reported inducible and tunable gene expression systems for use in *P. putida* KT2440 (Sathesh-Prabu et al., 2021) and showed that the efficiency rates of the glucose-inducible HexR/P_*zwfA*_, LA-inducible LvaR/P_*lvaA*_, 3-HP- and LA-inducible HpdR/P_*hpdH*_, and xylose-inducible XutR/P_*xutA*_ were comparable to that of conventional chemical-inducible systems. In contrast, in the present study, HexR/P_*zwfA*_, LvaR/P_*lvaA*_, and XutR/P_*xutA*_ were non-functional in *M. extorquens* AM1. This result may be attributed to the lack of transporters for glucose and xylose. Notably, LA, the native inducer of P_*lvaA*_, failed to induce the LvaR/P_*lvaA*_ system, but induced the HpdR/P_*hpdH*_ system. This finding suggests that the transcriptional regulator LvaR might be unexpressed or non-functional or that the LA-bound LvaR may not be able to form an active form of LvaR–DNA complex or recruit RNA polymerase for transcription. Moreover, the orthogonality of P_*hpdH*_ and P_*mmsA*_ of *P. putida* KT2440 has been demonstrated in *E. coli* and *Cupriavidus necator* (Hanko et al., 2017). In the present study, we showed the functional eGFP^+^ expression of the HpdR/P_*hpdH*_ and MmsR/P_*mmsA*_ systems in *M. extorquens* AM1. The HpdR/P_*hpdH*_ and MmsR/P_*mmsA*_ systems are involved in the catabolism of 3-HP in *Pseudomonas* species (Zhou et al., 2015; Hanko et al., 2017). Interestingly, the HpdR/P_*hpdH*_ system was found to be activated by two different inducers (3-HP and LA) in *M. extorquens* AM1. This result is in accordance with that of a recent study conducted on *P. putida* KT2440 (Sathesh-Prabu et al., 2021). The HpdR/P_*hpdH*_ system was tightly controlled, with a basal expression of 2%. The minimum requirement for the ligand to be functional and induce the HpdR/P_*hpdH*_ system is one carboxyl group, as present in 3-HP, propionate, butyrate, d-3-hydroxybutyrate, and l-3-hydroxybutyrate (Hanko et al., 2017). LA also contains one carboxyl group. The presence of a hydroxyl group at the β-position enhances regulator activity. To our knowledge, this is the first description of 3-HP- and LA-inducible systems in *M. extorquens* AM1.

The IPTG-inducible system is currently the most efficient method for the overexpression of recombinant proteins in many hosts of biotechnological interest, such as *E. coli* (Balzer et al., 2013; Briand et al., 2016), *P. putida* KT2440 (Calero et al., 2016), and *M. extorquens* AM1 (Carrillo et al., 2019). However, the extremely high level of transcription in the IPTG-inducible system resulted in a correspondingly high production of insoluble and inactive proteins and a high level of background expression (Balzer et al., 2013). In addition, it is not cost effective and is incompatible with scaling up on an industrial level. In contrast, the reported system was efficiently induced by the low-cost renewable LA, tightly controlled, and achieved a high level of expression relative to the IPTG-inducible system with a lower level of basal expression (without inducer).

Since the added LA was not degraded by strain MHRH01, a stable gene expression level can be envisaged. However, the addition of small doses of IPTG at multiple time points during cultivation is necessary to maintain an optimal concentration of IPTG for the efficient production of recombinant proteins (Hu et al., 2015). Furthermore, LA, when used as a substrate for biochemical production, can be efficiently utilized for both induction and production with a higher yield of the desired product. LA (a five-carbon compound) is a sustainable platform molecule and has been identified as one of the “top 12” promising sugar-based building blocks by the United States Department of Energy (Werpy and Petersen, 2004). LA can be obtained from renewable cellulosic biomass through acid-catalyzed dehydration and hydrolysis (Hayes and Becer, 2020). As LA contains both ketone and carboxylic acid functional groups, a wide range of valuable chemicals and fuels can be produced from it (Pileidis and Titirici, 2016). Moreover, low-cost LA is considered to be a promising organic intermediate for the synthesis of several chemicals such as polymers, plasticizers, fuels, resins, fragrances, pharmaceuticals, anti-freeze agents, and solvents (Bozell et al., 2000; Rackemann and Doherty, 2011; Pileidis and Titirici, 2016; Sathesh-Prabu and Lee, 2019; Cha et al., 2020; Hayes and Becer, 2020).

The system constructed here efficiently produced the reporter protein with a specific GFP production of 42 mg/g biomass, representing 10% of the total cell protein. The efficiency of our system was similar to that of the widely used promoter P_*mxaF*_ in *M. extorquens.* The strong natural promoter P_*mxaF*_ in *M. extorquens* produced 41–48 mg/g biomass of GFP at the shake-flask level, representing 8%–10% of the total cell protein. In addition, a maximum of 80 mg/g biomass of GFP (≈16% of the total cell protein) was obtained during the fed-batch cultivation of *M. extorquens* in a 20-L fermenter with a constant methanol concentration of 1.4 g/L for 50 h of cultivation (Bélanger et al., 2004). Thus, the LA-inducible system achieved an almost absolute P_*mxaF*_ efficiency. Other inducible promoter systems (P_*R/cmtO*_ and P_*R/tetO*_) were reported to have a maximum of 33% P_*mxaF*_ in *M. extorquens* AM1 (Chubiz et al., 2013). Moreover, the constructed LA-inducible system achieved 90%–94% efficiency of that of P_*L/O4*_, the recently developed IPTG-inducible system that is considered to be stronger than P_*mxaF*_ (Carrillo et al., 2019).

In summary, a renewable and eco-friendly LA-inducible and tunable gene expression system, HpdR/P_*hpdH*_, was evaluated for use in *M. extorquens* AM1. The system showed almost equal efficiency to the widely used strong promoters in *M. extorquens* AM1. The advantages of this system include the regulatable and tight control of the system, low cost, ease of LA induction, non-degradation of LA, and strain independence. Hence, the reported system is highly useful for expanding the biotechnological applicability of the potent strains of *M. extorquens* in C1 biorefinery.

## Data Availability

The original contributions presented in the study are included in the article/Supplementary Material. Further inquiries can be directed to the corresponding author.
