# Supplementary material for: Levulinic Acid-Inducible and Tunable Gene Expression System for Methylorubrum extorquens
Source: Front Bioeng Biotechnol. 2021 Dec 15;9:797020. doi: 10.3389/fbioe.2021.797020 (PMC8714952; doi:10.3389/fbioe.2021.797020)
Supplement: Supplementary file 1 [file DataSheet1.pdf]

Supplementary Table S1. Strains and plasmids used in this study

Strains & plasmids	Description	Reference
**Strains**		
E. coli		
DH10B	Cloning host (F^- *mcrA* Δ(*mrr-hsdRMS-mcrBC*) φ80*lacZ*ΔM15 Δ*lacX74 recA1 endA1 araD139* Δ(*ara-leu*)*7697 galU galK* λ^- *rpsL*(Str^R) *nupG*)	Lab stock
M. extorquens		
AM1	*M. extorquens* AM1; ATCC 14718	Lab stock
PA1	*M. extorquens* PA1; DSM 23939	Lab stock
CM4	*M. extorquens* CM4; KCTC 32005	Lab stock
DM4	*M. extorquens* DM4; DSM 6343	Lab stock
TK0001	*M. extorquens* TK0001; DSM 1337	Lab stock
ATCC55366	*M. extorquens* ATCC 55366	Lab stock
MHRZ01	AM1 harboring pMHRZ_eGFP$^+$	This study
MLRL01	AM1 harboring pMLRL_eGFP$^+$	This study
MHRH01	AM1 harboring pMHRH_eGFP$^+$	This study
MMRM01	AM1 harboring pMMRM_eGFP$^+$	This study
MXRX01	AM1 harboring pMXRX_eGFP$^+$	This study
MPLO4	AM1 harboring pMPLO4_eGFP$^+$	This study
PA1HRH01	PA1 harboring pMHRH_eGFP$^+$	This study
CM4HRH01	CM4 harboring pMHRH_eGFP$^+$	This study
DM4HRH01	DM4 harboring pMHRH_eGFP$^+$	This study
TK01HRH01	TK0001 harboring pMHRH_eGFP$^+$	This study
AT66HRH01	ATCC55366 harboring pMHRH_eGFP$^+$	This study
**Plasmids**		
pCM110_ P_{mxaF}_Fdh1	*M. extorquens* expression vector, P_{mxaF}, Tet^R	Jang et al., 2018
pCM110_$P_{L/O4}$	Derivative of pCM110, LacI/ $P_{L/O4}$, Tet^R	This study
pHRZ_eGFP$^+$	HexR/P_{zwf1} cloned into pPROBE_$P_{yqjFmut}$_eGFP$^+$	Sathesh-Prabu et al., 2021
pLRL_eGFP$^+$	LvaR/P_{lvaA} cloned into pPROBE_$P_{yqjFmut}$_eGFP$^+$	Sathesh-Prabu et al., 2021
pHRH_eGFP$^+$	HpdR/P_{hpdH} cloned into pPROBE_$P_{yqjFmut}$_eGFP$^+$	Sathesh-Prabu et al., 2021
pMRM_eGFP$^+$	MmsR/P_{mmsA} cloned into pPROBE_$P_{yqjFmut}$_eGFP$^+$	Sathesh-Prabu et al., 2021

pXRX_eGFP$^+$	XutR/P_{xutA} cloned into pPROBE_$P_{yqjFmut}$_eGFP$^+$	Sathesh-Prabu et al., 2021
pMHRZ_eGFP$^+$	HexR/P_{zwf1} cloned into pCM110_Fdh1	This study
pMLRL_eGFP$^+$	LvaR/P_{lvaA} cloned into pCM110_Fdh1	This study
pMHRH_eGFP$^+$	HpdR/P_{hpdH} cloned into pCM110_Fdh1	This study
pMMRM_eGFP$^+$	MmsR/P_{mmsA} cloned into pCM110_Fdh1	This study
pMXRX_eGFP$^+$	XutR/P_{xutA} cloned into pCM110_Fdh1	This study
pMPLO4_eGFP$^+$	LacI/$P_{L/O4}$ cloned into pCM110_Fdh1	This study

The plasmid pCM110_ P_{mxaF}_Fdh1 and strains AM1, PA1, CM4, DM4, TK0001, and ATCC55366 were kindly gifted by Prof. Yong Hwan Kim, Ulsan National Institute of Science and Technology.

Supplementary Table S2: Oligomers used in this study

Oligomers	Sequences (5′-3′)	Purpose
Pro-FP	tgcatgcctgcaggtcgactctagacaggaattggggatcggaag	To amplify the fragment "Terminator-Regulator/P*x*-eGFP^{+}" from the template plasmids to generate the following plasmids, pMHRZ_eGFP^{+}, pMLRL_eGFP^{+}, pMHRH_eGFP^{+}, pMMRM_eGFP^{+}, and pMXRX_eGFP^{+}
Pro-RP	gttagcagccctcgagtttggatccgtccaagctcagctaattaagc	
PLO4-FP	cgtttccaccgaattagcttgcatgcctgcaggtcgactctagatcac tgcccgctttccagtcggg	To amplify LacI and PLO4 to construct pCM110_$P_{L/O4}$
PLO4-RP	tcctgctgatgtgctcagtatcattgttatccgctcacatgtcaacacc gccagagataatttatcgcatgcaccattccttgcggcggc	
PLO4-RP2	caactcagcttcctttcgggctttgttagcagccggatccgtcagtgc gtcctgctgatgtgctcagtatcattg	
LacI/$P_{L/O4}$-FP	cgtttccaccgaattagcttgcatgcctgcaggtcgactctagatcac tgcccgctttccagtcggg	To amplify LacI_PLO4 from pCM110_$P_{L/O4}$ to construct pMPLO4_eGFP^{+}
LacI/$P_{L/O4}$-RP	cgacggatccatgtatatctccttcttaaagttaaacaaagtcagtgc gtcctgctgatg	
eGFP^{+}-FP	agatatacatggatccgtcgactgcagc	To amplify eGFP^{+} from pMHRH_eGFP^{+} to construct pMPLO4_eGFP^{+}
eGFP^{+}-RP	gttagcagccctcgagtttggatccgtccaagctcagctaattaa gcttatttg	

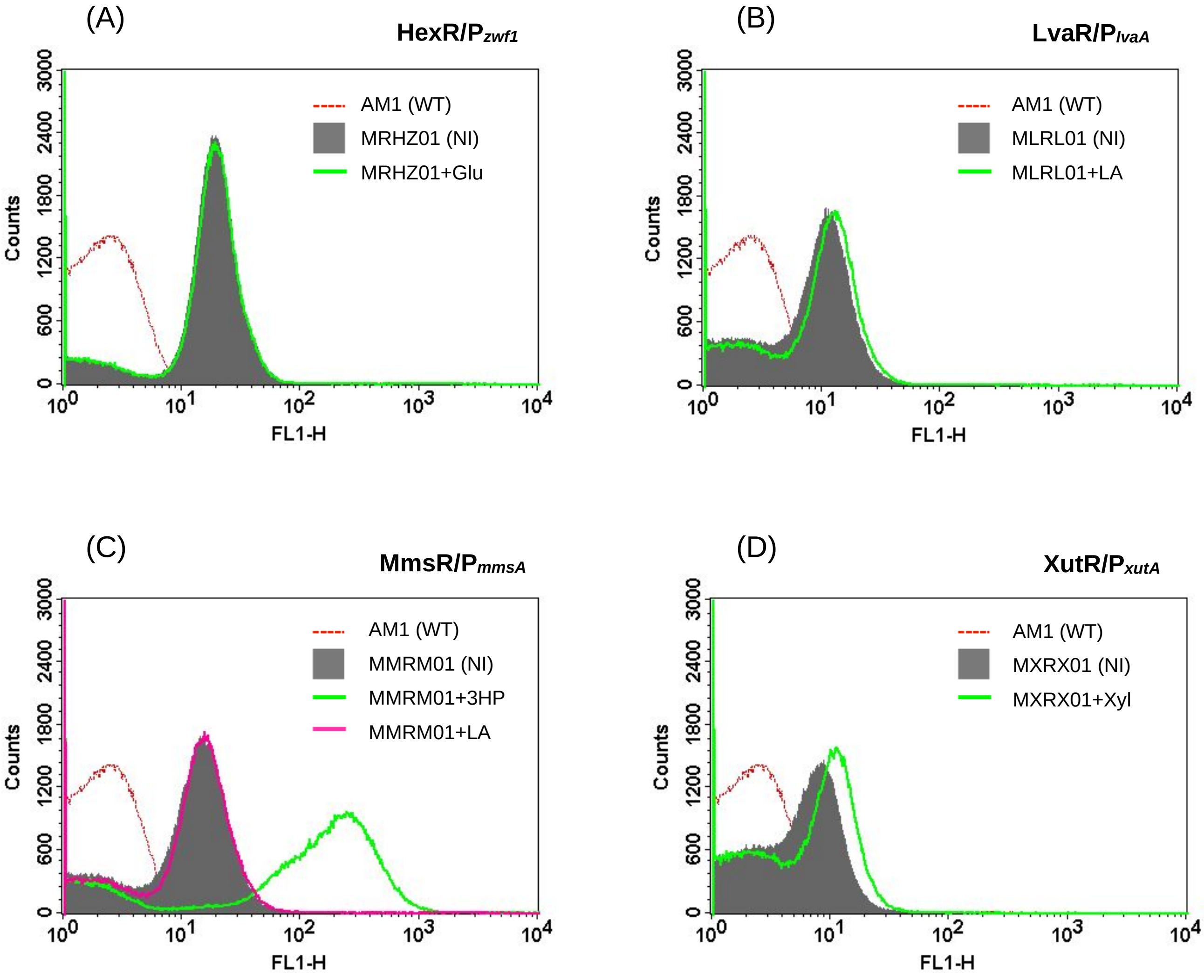

Supplementary Figure S1: Flow cytometric analysis of various inducible expression systems in *M. extorquens* AM1. (A) Flow cytometric analysis of the strain MHRZ01 harboring the HexR/P$_{zwf1}$ system. (B) Flow cytometric analysis of the strain MLRL01 harboring the LvaR/P$_{lvaA}$ system. (C) Flow cytometric analysis of the strain MMRM01 harboring the MmsR/P$_{mmsA}$ system. (D) Flow cytometric analysis of the strain MXRX01 harboring the XutR/P$_{xutA}$ system. The strains were cultivated with 10 mM solutions of their respective inducers. NI, not induced; Glu, glucose; LA, levulinic acid; 3HP, 3-hydroxypropionic acid; Xyl, xylose.

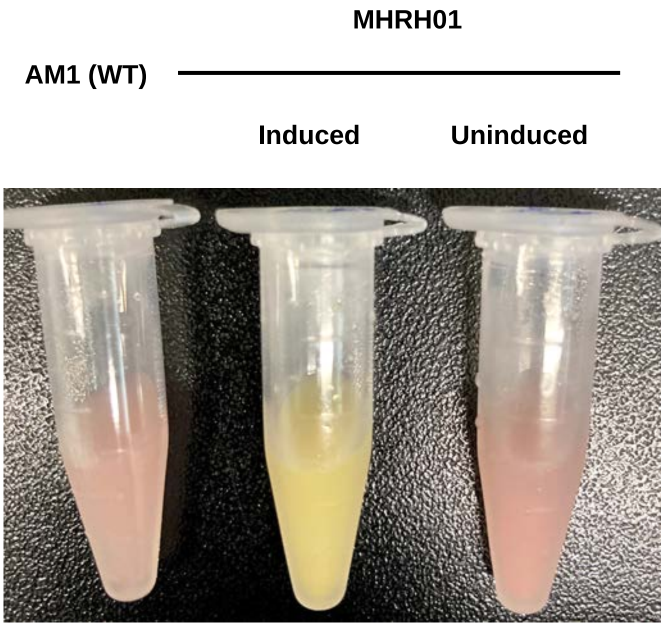

Supplementary Figure S2: Comparison of colors of the cultures. The strain MHRH01 cultivated with 10 mM LA showed green color, demonstrating a strong expression of GFP under the LA-inducible HpdR/P_{hpdH} system

References

Jang, J., Jeon, B. W., and Kim, Y. H. (2018). Bioelectrochemical Conversion of CO2 to Value-added Product Formate using Engineered *Methylobacterium extorquens*. *Sci. Rep.* 8:7211. doi:10.1038/s41598-018-23924-z.

Sathesh-Prabu, C., Tiwari, R., Kim, D., and Lee, S. K. (2021). Inducible and Tunable Gene Expression Systems for *Pseudomonas putida* KT2440. *Sci. Rep.* 11:18079. doi:10.1038/s41598-021-97550-7.
